# In This Issue

**Published:** 2001

## ALCOHOL-RELATED BIRTH DEFECTS—THE PAST, PRESENT, AND FUTURE

In 1994 *Alcohol Health & Research World* (now titled *Alcohol Research & Health*) last devoted a full issue to the topic of fetal alcohol syndrome (FAS) and other alcohol-related birth defects (ARBD). This article by Drs. Kenneth R. Warren and Laurie L. Foudin provides readers with information on how the field has advanced since 1994. In addition to tracing the development of the terminology used in the field, the authors describe the difficulties involved in determining the true prevalence of FAS and associated disorders; the mechanisms that may play a role in alcohol-derived fetal injuries; approaches to preventing drinking during pregnancy; and strategies for assisting people who have been born with alcohol-related birth defects. (pp. 153–158)

## ESTIMATING THE PREVALENCE OF FETAL ALCOHOL SYNDROME

Determining just how many infants are born each year with alcohol-related birth defects is a daunting task. In this article, Drs. Philip A. May and J. Phillip Gossage describe the primary methods used by researchers to estimate the prevalence of fetal alcohol syndrome (FAS), alcohol-related birth defects (ARBD), and alcohol-related neurodevelopmental disorders (ARND). The authors also report on the maternal risk factors associated with FAS and other alcohol-related birth disorders, such as advanced maternal age, low socioeconomic status, frequent binge drinking, and other social and psychological factors. (pp. 159–167)

## DRINKING PATTERNS AND ALCOHOL-RELATED BIRTH DEFECTS

Not all children whose mothers drank heavily during pregnancy have severe alcohol-related birth defects. Even in those children with full-blown fetal alcohol syndrome, the degree of impairment and related disorders may vary substantially. One factor contributing to this variability is the mother’s drinking pattern. Drs. Susan E. Maier and James R. West explore the hypothesis that a pattern of binge drinking—particularly during early pregnancy—is especially harmful to the fetus. Studies in both experimental animals and humans support this hypothesis. Animal studies demonstrate that bingelike exposure is associated with greater deficits (e.g., reduced brain growth) than is continuous exposure to alcohol. Long-term studies in humans have confirmed these findings. Children exposed to binge drinking prenatally show greater and more persistent deficits than children exposed to more continuous drinking patterns. ( pp. 168–174)

## MECHANISMS OF ALCOHOL-INDUCED DAMAGE TO THE DEVELOPING NERVOUS SYSTEM

Aplethora of mechanisms are responsible for alcohol’s deleterious effects on the developing fetus. Dr. Charles R. Goodlett and Ms. Kristin H. Horn review some of these factors, including the induction of cell death through processes called necrosis and apoptosis. Numerous factors can result in cell death, including a malfunction of the cell’s energy generators (i.e., the mitochondria) or buildup of lethal and highly reactive oxygen-containing molecules. Excessive nerve cell activity and disruptions in the developing brain’s chemical networks also can contribute to alcohol-induced brain damage. In addition, alcohol may interfere with the body’s growth factors, important molecules that regulate cell growth and survival. Given the wide variety of mechanisms affected by alcohol, it may not be possible to undo the harmful effects of drinking during pregnancy. Still, a better understanding of these mechanisms may help scientists devise ways of lessening alcohol’s impact on the developing fetus. (pp. 175–184)

## TERATOGENIC EFFECTS OF ALCOHOL ON BRAIN AND BEHAVIOR

Children prenatally exposed to alcohol may suffer a number of serious developmental deficits, including fetal alcohol syndrome (FAS) and the less severe fetal alcohol effects (FAE). Dr. Sarah N. Mattson, Ms. Amy M. Schoenfeld, and Dr. Edward P. Riley summarize the results of neuropsychological studies of alcohol’s effects on behavior in children with FAS or FAE. They found that those children typically have lower IQ scores, impairments in learning new information, deficits in higher-level cognitive abilities, psychosocial deficits, and problem behaviors. The authors explore how brain imaging techniques are helping researchers to better understand how these behavioral effects coincide with changes in brain structure. (pp. 185–191)

## THE EFFECTS OF PRENATAL ALCOHOL EXPOSURE ON EXECUTIVE FUNCTIONING

The term “executive functioning” refers to the cognitive functions involved in planning and guiding behavior. These functions can be classified into cognition-based and emotion-related executive function. According to Drs. Piyadasa W. Kodituwakku, Wendy Kalberg, and Philip A. May, these functions may be particularly impaired in people prenatally exposed to alcohol and may contribute to some of the behavioral problems observed in alcohol-exposed children and adults (e.g., difficulty in understanding the social consequences of behavior). Alcohol-related abnormalities in certain brain structures and in the connections among brain regions may account for at least some of these deficits in executive functioning. (pp. 192–198)

## FETAL ALCOHOL EXPOSURE AND ATTENTION

Children with prenatal alcohol exposure, especially fetal alcohol syndrome (FAS), often are said to exhibit behaviors consistent with attention deficit hyperactivity disorder (ADHD). However, not all studies of alcohol-exposed children support the association between ADHD and FAS. In this short sidebar article, Dr. Claire D. Coles summarizes recent studies comparing ADHD children with those diagnosed with either FAS or partial FAS. The findings suggest that FAS children do not necessarily have the same neurocognitive deficits as those seen in children diagnosed with ADHD. Dr. Coles contends that such findings indicate that understanding the relationship between prenatal alcohol exposure and attention will require a multifaceted approach that moves beyond ADHD. She suggests, for example, that researchers evaluating children’s development should consider the many factors that affect development, such as caregiving, as well as the cognitive processes and other components that comprise behavior. (pp. 199–203)

## ALCOHOL SCREENING INSTRUMENTS FOR PREGNANT WOMEN

Because even low levels of prenatal alcohol exposure can negatively affect the developing fetus, identifying women who drink during pregnancy is an important step in preventing alcohol-related birth defects. Still, as discussed by Dr. Grace Chang, obstetricians inconsistently screen their pregnant patients for alcohol use. To help improve alcohol screening during pregnancy, a number of short screening questionnaires have been developed for use with pregnant women. Dr. Chang reviews these screening instruments and discusses their effectiveness and use. (pp. 204–209)

## MARKERS TO DETECT DRINKING DURING PREGNANCY

Women who drink alcohol during pregnancy often deny or minimize their drinking when asked about it. A number of biological markers, or biomarkers, are being developed to provide a definitive laboratory test to detect alcohol use among pregnant women. Biomarkers typically signal events, or changes, in the body. A biomarker that could detect alcohol use during pregnancy would allow earlier identification of alcohol-exposed children, and thereby improve intervention efforts. Dr. Cynthia F. Bearer reviews the development and use of biomarkers in general and reports on potential new biomarkers for detecting alcohol use during pregnancy. These markers not only would improve intervention for alcohol-exposed infants, but also would aid in identifying women at risk for alcohol use during subsequent pregnancies, help to detect underreporting of alcohol use during pregnancy, and facilitate research on the relationship between different levels of alcohol exposure and alcohol-related birth defects. (pp. 210–218)

## MOTIVATIONAL INTERVENTIONS IN PRENATAL CLINICS

Pregnant women who drink at levels that present the greatest risk often have not received prevention measures, according to Dr. Nancy Sheehy Handmaker and Ms. Paula Wilbourne. The authors review a wide variety of studies to show how caregivers can help pregnant women reduce their drinking. Motivational interviewing, in particular, is proving successful in prompting some women to reduce or eliminate drinking during pregnancy, according to the authors. They also outline a stepped-care approach that enables practitioners to intervene to prevent drinking during pregnancy, while minimizing costs to the patient and demands for limited clinic resources. (pp. 219–229)

## ALCOHOL’S EFFECT ON LACTATION

In many cultures, folklore holds that nursing women should drink alcohol to encourage production and release of their breast milk as well as to relax themselves and their infants. Scientific studies, however, have not supported these beliefs, reports Dr. Julie Mennella. Such studies show that infants actually consumed less milk after their mothers drank alcohol because maternal milk production was modestly reduced. In addition, some of the alcohol is transferred to the milk and ingested by the infant. Rather than relax the infant—as suggested by the folklore—alcohol actually may disrupt the infant’s sleep. The author reviews the effects of alcohol in breast milk, including how regular alcohol exposure through breast milk may slightly delay an infant’s gross motor development. She also explores factors that may place infants at particular risk for experiencing problems from exposure to alcohol in breast milk. (pp. 230–234)

